# Analysis of endoscopic and pathological features of 6961 cases of gastric cancer

**DOI:** 10.1038/s41598-024-58018-6

**Published:** 2024-03-26

**Authors:** Junhui Lu, Qing Wang, Hezhao Zhang, Jingwei Liu, Jinnan Ren, Jing Fan, Jingwen Gong, Yue Sui, Xing Chen

**Affiliations:** 1 Faculty of Graduate Studies, Shanxi Medical University, Taiyuan City, 030000 Shanxi Province China; 2 Department of Gastroenterology, First Hospital of Shanxi Medical University, Jie Fang South Road No.85, Taiyuan City, 030000 Shanxi Province China; 3 Department of Gastroenterology, Shanxi Provincial People’s Hospital, Taiyuan City, 030000 Shanxi Province China

**Keywords:** Cancer screening, Gastrointestinal cancer

## Abstract

Gastric cancer (GC) stage and tissue differentiation affect treatment efficacy and prognosis, highlighting the importance of understanding the risk factors that affect these parameters. Therefore, this study analyzed risk factors affecting the GC stage and differentiation and the relationships between the cancer site and the sex and age of the patient. We collected clinical data from 6961 patients with GC, including sex, age, endoscopic lesion location, and pathological differentiation. Patients were grouped based on GC stage (early or advanced), differentiation (well or poorly differentiated), and lesion site (upper stomach [cardia and fundus], middle stomach [gastric body], and lower stomach [gastric antrum]). Differences in sex, age, location, stage, and degree of differentiation were assessed based on these groupings. Univariate analysis revealed that the disease location and differentiation significantly differed based on the GC stage (*P* < 0.05), whereas sex, age, site, and stage significantly differed based on GC differentiation (*P* < 0.05). A multivariate analysis confirmed these factors as independent risk factors affecting GC. Moreover, lesion sites significantly differed between sexes (*P* < 0.05) and among age groups (*P* < 0.05). Although the effects of family history, lifestyle, and *Helicobacter pylori* infection status of the patients were not considered, this single-center retrospective study established independent risk factors for GC.

*Trial registration* ChiCTR2200061989.

## Introduction

Cancer affects longevity and quality of life and is a leading cause of death. In 2020, approximately 1.089 million new gastric cancer (GC) cases worldwide and approximately 769,000 deaths from GC were reported worldwide. China accounted for, 43.9% of these new cases and 48.6% of reported deaths, ranking first worldwide^1^, which highlights the importance of GC prevention and control in China. The onset of GC is insidious, and most patients with early GC do not exhibit typical clinical manifestations. Consequently, by the time clinical symptoms manifest, the disease has progressed to an advanced stage. Unfortunately, the quality of life and prognosis of patients with advanced GC are poor, with a five-year overall survival rate of 35.1% in China and less than 30% in Europe^2,3^.

Notably, the GC stage affects treatment and prognosis^4,5^. The prognoses of early and advanced GC differ significantly, with the five-year survival rate of patients with early GC exceeding 96%^6,7^, whereas it is only 15% for those with advanced GC^8^. Furthermore, the degree of differentiation in GC tissues affects the prognosis; as the degree of differentiation decreases, the risk of death gradually increases. Specifically, as GC tissue differentiation decreases stepwise from high to low, the risk of death increases three-fold for each step^9^. Therefore, understanding the risk factors that affect the GC stage and degree of differentiation is essential. The present study aimed to analyze the risk factors affecting GC stage and degree of differentiation, as well as the effects of age, sex, and incidence site on these two parameters.

## Patients and methods

### Patients

We enrolled patients diagnosed with GC by gastroscopy, with pathological confirmation at the Shanxi Cancer Registration Center from January 2017 to December 2018. The Ethics Committee of the Shanxi Cancer Hospital approved this study (2,019,091). All patients provided written informed consent, and the trial passed the clinical trial registration process (ChiCTR2200061989). The study was performed in accordance with the principles of the Declaration of Helsinki.

Patients who underwent gastroscopy for the first time and had pathologically confirmed GC after a biopsy were included. Furthermore, all included participants agreed to undergo a detailed examination using the linkage imaging mode of the gastroscope (LASEREO EG L590ZW, FUJIFILM, Tokyo, Japan). Those with incomplete clinical, endoscopic, and pathological data, multiple GC lesions, secondary GC after remnant stomach surgery, and other primary or secondary cancers were excluded.

### Definition

Early gastric cancer refers to tumors that are confined to the mucosal and submucosal layers, regardless of the presence or absence of lymph node metastasis^10^. Advanced gastric cancer is characterized by tumor invasion beyond the submucosal layer into deeper layers, which may include penetration into the muscularis propria, serosa, or the occurrence of lymph node metastasis and/or distant metastasis^10^. In this study, the early gastric cancer specimens include a portion of those resected by endoscopic submucosal dissection (ESD) and a portion from gastrectomy specimens. Advanced gastric cancer specimens include those from gastrectomy as well as those diagnosed based on endoscopic appearance and biopsy pathology results, due to a subset of patients opting against surgical treatment.

### Data collection and groupings

We collected data on sex, age, endoscopic lesion location, and pathological differentiation. Patients were grouped based on GC stage (early or advanced), lesion site (upper stomach [cardia and fundus], middle stomach [gastric body], and lower stomach [gastric antrum]), and differentiation status (well-differentiated [well and moderately differentiated] and poorly differentiated [poorly differentiated, mucinous adenocarcinoma, and signet ring cell carcinoma]).

### Statistical analyses

The risk factors affecting GC stage and differentiation degree, as well as differences between age, sex, and incidence site groups, were analyzed using SPSS 22.0 (IBM Corp., Armonk, NY, USA). Qualitative data are presented as the number of cases and percentages, and the *c*^2^ test was used to compare two groups. Influencing factors were analyzed by logistic regression. All variables with *P* < 0.1 in the univariate analysis were used in the multivariate analysis. Statistical significance was set at *P* < 0.05.

### Ethical approval

The Ethics Committee of the Shanxi Cancer Hospital approved this study (2019091). All patients provided written informed consent, and the trial passed the clinical trial registration process (ChiCTR2200061989).

## Results

### Patient demographics

We included 6961 patients diagnosed with GC. Table 1 presents the baseline characteristics of these patients.

**Table 1:** Baseline characteristics.

Factors	N (%)
Sex	
Male	5653 (81.21)
Female	1308 (18.79)
Age (years)	
Range	19–93
Average (mean ± standard deviation)	61.59 ± 9.83
< 40	163 (2.34)
40–49	584 (8.39)
50–59	1895 (27.22)
60–69	2929 (42.08)
70–79	1218 (17.5)
≥ 80	172 (2.47)
Lesion location	
Upper	3456 (49.65)
Middle	2237 (32.14)
Lower	1268 (18.22)
Stage	
Early	942 (13.53)
Advanced	6019 (86.47)
Pathological differentiation	
Well-differentiated	2516 (36.14)
Poorly differentiated	4445 (63.86)

### GC stage risk factors

The univariate analysis identified significant differences in the GC stage based on the lesion site (*P* < 0.05; Table 2). The incidence rate of advanced GC significantly differed between the lower and upper and middle locations (*P* < 0.05), whereas it did not differ between the upper and middle locations. Furthermore, the degree of differentiation significantly differed between GC stages (*P* < 0.05), whereas sex and age did not (*P* > 0.05). The multivariate analysis identified disease location and degree of differentiation as significant variables (*P* < 0.05), and thus, as independent risk factors affecting the GC stage (Table 3).

**Table 2:** Univariate analysis of gastric cancer stages.

Factors	Early GC N (%)	Advanced GC N (%)	*c*^2^	*P*-value
Sex			2.061	0.151
Male	781 (13.82)	4872 (86.18)		
Female	161 (12.31)	1147 (87.69)		
Age* (years)			0.010	0.909
< 40	18 (11.04)	145 (88.96)		
40–49	75 (12.84)	509 (87.16)		
50–59	261 (13.77)	1634 (86.23)		
60–69	411 (14.03)	2518 (85.97)		
70–79	155 (12.73)	1063 (87.27)		
≥ 80	22 (12.79)	150 (87.21)		
Pathological differentiation			995.113	< 0.001
Well-differentiated	773 (30.72)	1743 (69.28)		
Poorly differentiated	169 (3.80)	4276 (96.20)		
Lesion location			79.330	< 0.001
Upper	422 (12.21)	3034 (87.79)		
Middle	251 (11.22)	1986 (88.78)		
Lower	269 (21.21)	999 (78.79)^a,b^		

*Analyzed by trend test; ^a^P < 0.05 compared with the upper location; ^b^*P* < 0.05 compared with the middle location. GC: Gastric cancer.

**Table 3:** Multivariate analysis of gastric cancer stages.

Factors	*β*	SE	Wald	*P*-value	OR	OR 95% CI	
						Upper limit	Lower limit
Pathological differentiation	2.470	0.091	739.451	< 0.001	11.825	9.897	14.130
Lesion location	–	–	93.120	< 0.001	–	–	–
Upper	–	–	–	–	1.000	–	–
Middle	–0.118	0.091	1.667	0.197	0.889	0.743	1.063
Lower	–0.904	0.096	88.147	< 0.001	0.405	0.335	0.489

*CI* Confidence interval,* OR* Odds ratio,* SE* Standard error.

### GC differentiation risk factors

In the univariate analysis, sex, age, location, and stage significantly differed based on the differentiation status (*P* < 0.05; Table 4). Specifically, the middle and upper location incidence rates significantly differed (*P* < 0.05), as did those for the lower, and upper and middle locations (both *P* < 0.05). The multivariate analysis identified sex, age, location, and stage as significant variables (*P* < 0.05), and thus, independent risk factors affecting GC differentiation (Table 5).

**Table 4:** Univariate analysis of gastric cancer differentiation.

Factors	Well-differentiated N (%)	Poorly differentiated N (%)	*c*^2^	*P*-value
Sex			50.045	< 0.001
Male	2154 (38.10)	3499 (61.90)		
Female	362 (27.68)	946 (72.32)		
Age* (years)			42.681	< 0.001
< 40	20 (12.27)	143 (87.73)		
40–49	150 (25.68)	434 (74.32)		
50–59	695 (36.68)	1200 (63.32)		
60–69	1124 (38.37)	1805 (61.63)		
70–79	455 (37.36)	763 (62.64)		
≥ 80	72 (41.86)	100 (58.14)		
Stage			995.113	< 0.001
Early	773 (82.06)	169 (17.94)		
Progressive	1743 (28.96)	4276 (71.04)		
Lesion location			41.649	< 0.001
Upper	1367 (39.55)	2089 (60.45)		
Middle	697 (31.16)	1540 (68.84)^a^		
Lower	452 (35.65)	816 (64.35)^a,b^		

*Analyzed by trend test; ^a^*P* < 0.05 compared with the upper location; ^b^*P* < 0.05 compared with the middle location.

**Table 5:** Multivariate analysis of gastric cancer differentiation.

Factors	*β*	SE	Wald	*P*-value	OR	OR 95% CI	
						Upper limit	Lower limit
Sex	0.439	0.075	34.716	< 0.001	1.551	1.340	1.795
Age (years)	–	–	57.365	< 0.001	–	–	–
< 40	–	–	–	–	1.000	–	–
40–49	− 0.942	0.284	11.010	0.001	0.390	0.224	0.680
50–59	− 1.450	0.270	28.894	< 0.001	0.235	0.138	0.398
60–69	− 1.509	0.268	31.682	< 0.001	0.221	0.131	0.374
70–79	− 1.493	0.273	29.984	< 0.001	0.225	0.132	0.384
≥ 80	− 1.699	0.313	29.484	< 0.001	0.183	0.099	0.338
Stage	2.500	0.092	734.15	< 0.001	12.182	10.167	14.597
Lesion location	–	–	36.618	< 0.001	–	–	–
Upper	–	–	–	–	1.000	–	–
Middle	0.322	0.063	26.442	< 0.001	1.379	1.220	1.559
Lower	0.358	0.078	21.127	< 0.001	1.431	1.228	1.667

CI: Confidence interval; OR: Odds ratio; SE: Standard error.

### GC location comparisons

Lesion location significantly differed between sexes (*P* < 0.05; Table 6). Specifically, the male-to-female ratio significantly differed between the upper and lower GC sites (*P* < 0.001), suggesting that men had a higher incidence rate of upper GC than GC in other sites. However, the male-to-female ratios did not differ between the middle and lower sites (*P* > 0.05). Moreover, disease location significantly differed among age groups (*P* < 0.05; Table 7).

**Table 6:** Relationships between gastric cancer location and sex.

Factors	Male N (%)	Female N (%)	*c*^2^	*P*-value
Lesion location			44.051	< 0.001
Upper	2913 (84.29)	543 (15.71)		
Middle	1762 (78.77)	475 (21.23)^a^		
Lower	978 (77.13)	290 (22.87)^a^		

^a^*P* < 0.05 compared with the upper location.

**Table 7:** Relationships between gastric cancer location and age (years).

Factors	< 40 N (%)	40–49 N (%)	50–59 N (%)	60–69 N (%)	70–79 N (%)	≥ 80 N (%)	*c*^2^	*P*-value
Lesion location							170.229	< 0.001
Upper	32 (0.93)	192 (5.56)	901 (26.07)	1556 (45.02)	676 (19.56)	99 (2.86)		
Middle	85 (3.8)	235 (10.51)	636 (28.43)	894 (39.96)	348 (15.56)	39 (1.74)^a^		
Lower	46 (3.63)	157 (12.38)	358 (28.23)	479 (37.78)	194 (15.30)	34 (2.68)^a^		

^a^P < 0.05 compared with the upper location.

## Discussion

GC is a gastrointestinal tumor with significant implications for human health. Early stages of GC typically lack clinical manifestations; therefore, most patients have progressed to the mid and late stages by the time of diagnosis, resulting in a poor quality of life and prognosis. Notably, Japan, the United States, and South Korea exhibit high early GC diagnosis rates exceeding 70%, approximately 70%, and exceeding 50%, respectively. In contrast, China and Europe exhibit a considerably lower early GC diagnosis rate of less than 10%^11–13^ contributing to a higher incidence of advanced GC at the time of diagnosis. Therefore, understanding the risk factors for advanced GC is crucial.

In the present study, our univariate analysis identified onset location and degree of differentiation as GC stage risk factors, which were subsequently confirmed as independent risk factors affecting GC stage in our multivariate analysis. These results are consistent with those of Yu et al. who reported that stage and degree of differentiation were independent risk factors for GC prognosis^4^. Similarly, Smyth et al. reported that poor differentiation was a risk factor for GC^10^. In the present study, the risk of poorly differentiated GC developing into advanced GC was 11.83 times higher than that of well-differentiated GC. Moreover, advanced GC was 2.47 times more likely to develop in the upper part of the stomach than in the lower part. Therefore, poorly differentiated GC and lesions in the upper stomach are more likely to progress into advanced GC. Consequently, clinical endoscopists should prioritize observation of the upper stomach, such as by using linked imaging technology or magnifying endoscopic observation, and become adept at recognizing the endoscopic appearance of poorly differentiated GC to decrease instances of missed diagnoses and improve early diagnosis rates, thereby prolonging patient survival^14^.

In addition, our results indicated that regardless of early or advanced GC, the most common GC site was the upper stomach. However, more patients exhibited early GC in the lower stomach than in the middle stomach, and more patients exhibited advanced GC in the middle than in the lower stomach. This result may be attributed to the fact that the middle stomach is more likely to be missed during an examination compared to the lower stomach. Nonetheless, this trend requires further investigation.

GC differentiation is an independent risk factor affecting GC stage and survival; therefore, analyzing the risk factors affecting GC differentiation is necessary. Our univariate analysis indicated that sex, age, location, and lesion stage affected GC differentiation, and all these parameters were confirmed as independent risk factors in the multivariate analysis. Furthermore, 81.21% of patients with GC in the present study were men, a significantly higher percentage than that of women. This result may be attributed to the higher prevalence of smoking and drinking among men than among women^15^.

Previous studies have reported associations between well-differentiated GC and older age, male sex, and earlier tumor stage, and between poorly differentiated GC and younger age, female sex, and advanced tumor stage^16^. Consistent with these findings, in the present study, women had a higher risk of poorly differentiated GC than men, and the risk of poorly differentiated GC decreased with age. Moreover, we demonstrated that regardless of lesion location, more patients had poorly differentiated GC. Notably, more patients with early GC had well-differentiated tumors, whereas more patients with advanced GC had poorly differentiated tumors. However, our sample size may not be sufficiently large to prevent bias, and it remains unknown if patients with poorly differentiated GC were more likely to have had a previously missed diagnosis, warranting further investigation.

In the present study, lesion location was an independent risk factor affecting GC stage; thus, group comparisons based on the disease site provide valuable insights. We also demonstrated that the incidence site significantly differed between sexes. Specifically, the prevalence rates of upper, middle, and lower GC in men were 84.29, 78.77, and 77.13%, respectively, higher than those in women (15.71%, 21.23%, and 22.87%, respectively). As a result, the GC incidence rates in the upper, middle, and lower stomach were 5.37, 3.71, and 3.37 times higher in men than in women, respectively. Additionally, in men, the incidence rate of upper stomach GC was higher than that in other parts of the stomach. These differences may be related to various levels of exposure to risk factors. For instance, a considerably higher portion of men smoke and drink alcohol than women, significantly increasing their risk of upper stomach GC^15–20^. Additionally, some studies have suggested that female estrogen is associated with a decreased GC incidence, which may also offer an explanation for the observed higher incidence of GC in men than in women in the present study^21,22^.

In the present study, GC location significantly differed among age groups. Of those aged 50–80 years, 90.65% had upper GC. Furthermore, of those aged 40–80 years, 94.46% had middle GC and 93.96% had lower GC. The incidence of GC remains low until the age of 40 years and then increases rapidly, peaking in both sexes after the age of 80^23,24^. However, there is no uniform age at which GC screening is initiated across countries. Several European countries and the UK endoscopy guidelines recommend that patients with intestinal metaplasia, as well as those with a family history of gastric cancer, incomplete-type intestinal metaplasia, or persistent *Helicobacter pylori*-associated gastritis, should undergo endoscopic surveillance every 3 years, whereas GC screening of asymptomatic individuals is not recommended^25–28^. Most Asian countries initiate GC screening between the ages of 40 and 45 years^29^, whereas the Japanese guidelines recommend GC screening starting at 50 years^30^. In the present study, over 10% of middle and lower GCs were detected in patients aged 40–50 years. These results highlight the need for appropriate measures to enhance awareness among the general population, conducting more early gastroscopies to rule out GC, and lowering the screening age to 40 years.

This study had several limitations that should be acknowledged. First, this was a retrospective single-center study with a small sample size; thus, our results require further confirmation in a more extensive study across multiple centers. Second, the effect of a family history of GC on differentiation and staging could not be assessed. Third, the cancer registration database did not include data on H. pylori infection status and eradication history; thus, we could not evaluate the effects of H. pylori on GC differentiation and staging. Finally, this study did not analyze the effects of smoking, drinking, diet, or other factors on GC differentiation and staging.

## Conclusion

This study demonstrated that the onset site and degree of differentiation were independent risk factors for GC stage, and sex, age, disease site, and lesion stage were independent risk factors for GC differentiation. Furthermore, GC location (upper, middle, or lower stomach) differed by age, and the prevalence of GC was higher in men than in women, especially in the upper stomach. Poorly differentiated GC and lesions in the upper stomach are more likely to progress into advanced GC; therefore, screening below the age of 40 years should be performed as an appropriate measure.

## Data Availability

The datasets used and/or analyzed during the current study are available from the corresponding author upon reasonable request.

## References

[1] Sung H (2021). Global cancer statistics 2020: GLOBOCAN estimates of incidence and mortality worldwide for 36 cancers in 185 countries. CA Cancer J. Clin..

[2] Zeng H (2018). Changing cancer survival in China during 2003–15: A pooled analysis of 17 population-based cancer registries. Lancet Glob. Health.

[3] De Angelis R (2014). Cancer survival in Europe 1999–2007 by country and age: Results of EUROCARE–5-a population-based study. Lancet Oncol..

[4] Yu H (2020). Association of ABO blood groups and risk of gastric cancer. Scand. J. Surg..

[5] Japanese Gastric Cancer Association (2017). Japanese gastric cancer treatment guidelines 2014 (ver. 4). Gastric Cancer.

[6] Katai H (2018). Five-year survival analysis of surgically resected gastric cancer cases in Japan: a retrospective analysis of more than 100,000 patients from the nationwide registry of the Japanese Gastric Cancer Association (2001–2007). Gastric Cancer.

[7] Gu L (2019). Comparison of long-term outcomes of endoscopic submucosal dissection and surgery for early gastric cancer: A systematic review and meta-analysis. J. Gastrointest. Surg..

[8] Lim SY (2020). Abdominal drainage in the prevention and management of major intra-abdominal complications after total gastrectomy for gastric carcinoma. J. Gastric Cancer..

[9] Hu X (2009). Progression and prognosis of gastric stump cancer. J. Surg. Oncol..

[10] Smyth EC (2020). Gastric cancer. Lancet.

[11] Ito Y (2021). Determinant factors on differences in survival for gastric cancer between the United States and Japan using nationwide databases. J. Epidemiol..

[12] Chiu PWY (2019). An Asian consensus on standards of diagnostic upper endoscopy for neoplasia. Gut.

[13] Chapelle N (2016). Early gastric cancer: Trends in incidence, management, and survival in a well-defined French population. Ann. Surg. Oncol..

[14] Lu JH (2023). Evaluation of the detection rate of high-grade gastric intraepithelial neoplasia using linked color imaging and white light imaging. Exp. Ther. Med..

[15] Nagel G (2018). Air pollution and incidence of cancers of the stomach and the upper aerodigestive tract in the European Study of Cohorts for Air Pollution Effects (ESCAPE). Int. J. Cancer.

[16] Feng F (2018). Prognostic value of differentiation status in gastric cancer. BMC Cancer.

[17] Onochi K (2019). Risk factors linking esophageal squamous cell carcinoma with head and) neck cancer or gastric cancer. J. Clin. Gastroenterol..

[18] Ma K (2017). Alcohol consumption and gastric cancer risk: A meta-analysis. Med. Sci. Monit..

[19] Millwood IY (2013). Alcohol consumption in 0.5 million people from 10 diverse regions of China: Prevalence, patterns and socio-demographic and health-related correlates. Int. J. Epidemiol..

[20] Shah SC (2020). Low baseline awareness of gastric cancer risk factors amongst at-risk multiracial/ethnic populations in New York City: Results of a targeted, culturally sensitive pilot gastric cancer community outreach program. Ethn. Health.

[21] Wang X (2017). Effects of 17β-estradiol and tamoxifen on gastric cancer cell proliferation and apoptosis and ER-α36 expression. Oncol. Lett..

[22] Zhao B (2019). Prognostic significance of tumour infiltration growth pattern in patients with advanced gastric cancer. J. Clin. Pathol..

[23] Kim YK (2012). Diagnostic accuracy and sensitivity of diffusion-weighted and of gadoxetic acid-enhanced 3-T MR imaging alone or in combination in the detection of small liver metastasis (≤ 1.5 cm in diameter). Invest. Radiol..

[24] Fock KM (2014). Review article: the epidemiology and prevention of gastric cancer. Aliment. Pharmacol. Ther..

[25] Lordick F (2022). Gastric cancer: ESMO Clinical Practice Guideline for diagnosis, treatment and follow-up. Ann. Oncol..

[26] Banks M (2019). British Society of Gastroenterology guidelines on the diagnosis and management of patients at risk of gastric adenocarcinoma. Gut.

[27] Cubiella J (2021). Gastric cancer screening in low incidence populations: position statement of AEG, SEED and SEAP. Gastroenterol. Hepatol..

[28] Delgado-Guillena PG (2021). Gastroenterologists’ attitudes on the detection and management of gastric premalignant conditions: Results of a nationwide survey in Spain. Eur. J. Cancer Prev..

[29] Kang YK (2017). Nivolumab in patients with advanced gastric or gastro-oesophageal junction cancer refractory to, or intolerant of, at least two previous chemotherapy regimens (ONO-4538-12, ATTRACTION-2): a randomized, double-blind, placebo-controlled, phase 3 trial. Lancet.

[30] Bang YJ (2012). Adjuvant capecitabine and oxaliplatin for gastric cancer after D2 gastrectomy (Classic): A phase 3 open-label, randomized controlled trial. Lancet.

